# Coping Strategies of Southern Italian Women Predict Distress Following Breast Cancer Surgery

**DOI:** 10.5964/ejop.v11i2.908

**Published:** 2015-05-29

**Authors:** Rossana De Feudis, Tiziana Lanciano, Stefano Rinaldi

**Affiliations:** a Psycho-Oncology, Breast Unit, “San Paolo” Hospital ASL BA, Bari, Italy; b Department of Education, Psychology, Communication, University of Bari Aldo Moro, Bari, Italy; c General Surgery Unit, Breast Unit, “San Paolo” Hospital of ASL BA, Bari, Italy; Warren Alpert Medical School, Brown University, Providence, USA; Department of Psychology, University of Toledo, Toledo, Spain

**Keywords:** breast cancer, breast surgery, coping, psychological distress, depression, anxiety

## Abstract

The present study was aimed at investigating the role of coping strategies in predicting emotional distress following breast cancer, over and above the illness severity, operationalized in terms of the type of surgery performed. In order to achieve this goal, two groups of newly diagnosed breast cancer women were selected and compared on the basis of the type of surgical treatment received. A subsample of 30 women with quadrantectomy and sentinel lymph-node biopsy (SLNB) and a subsample of 31 patients with mastectomy and axillary dissection (MAD) filled in the Brief Cope scale and Hospital Anxiety and Depression Scale. Summarizing, results showed that emotional support, venting, and humor explained a statistically significant increment of variance in psychological distress indices. Implication for clinical practice and future research were discussed.

Screening for emotional distress is becoming increasingly common in cancer care (Gandubert et al., 2009; Grassi et al., 2013; Holland, 2013; Schou, Ekeberg, Karesen, & Sorensen, 2008; Vodermaier, Linden, & Siu, 2009), since the new paradigm of taking into consideration the patients’ emotions, as well as their family relationships, as primary healthcare issues is gaining more and more consent in the medical domain (Engel, 1977; Seaburn, Lorenz, Gunn, Gawinski, & Mauksch, 1996).

The high incidence of breast cancer - 94.2 yearly rate (Age Standardized European Rate per 100,000) of EU women (118.0 of Italian women; Ferlay et al., 2013) - indicates the extent of this serious illness as a social problem. Many studies have demonstrated the pervasive, negative influence of breast cancer diagnosis upon quality of life (Carlson & Bultz, 2003; Mehnert & Koch, 2007; Montazeri, 2008; Henselmans et al., 2010). The short and long term emotional reactions, subsequent to the illness, may cause psychic suffering leading to serious psychological and health problems, such as chronic anxiety, depression, difficulties in sexual and social life (Burgess et al., 2005; Burke & Kissane, 1998; De Feudis, 2004; Holland, 2013). In addition, physical symptoms, motivation and participation to treatment can be affected by the presence of psychological disturbances (Burke & Kissane, 1998; Spiegel, 1997). Furthermore, the increasing survival rate has induced to consider the quality of life of women years after diagnosis and treatment. In fact, although recovered from cancer, relational difficulties, emotional dissatisfaction, underfunctioning, compared to personal and socio-family expectations, may persist (Andersen et al., 2008; Bleiker, Pouwer, van der Ploeg, Leer, & Adèr, 2000; Bower, 2008; Shelby, Golden-Kreutz, & Andersen, 2008; Spiegel, 1997).

Modern psychosocial programs for cancer patients, therefore, are aimed at detecting early signs of distress that can provide cues into the patient’s way of coping with the illness and orient the clinician towards adequate interventions (Carver, Scheier, & Weintraub, 1989; Glinder, Beckjord, Kaiser, & Compas, 2007; Stanton & Snider, 1993; Stanton et al., 2000). According to this theoretical and empirical evidence, each woman admitted to the Breast Unit at “San Paolo” Hospital of ASL BA, in Bari (Italy) is given the opportunity of a psychological consultation two days after surgery^i^.

Vos and colleagues (2004) found that in the period shortly after surgery, coping style, especially illness-specific coping, is highly relevant for psychosocial adjustment. The more women used an emotion-focused way of coping, the more distress and the less vitality was experienced. They also found that women who had breast-conserving treatment perceive their illness as less threatening than women who had mastectomy; in addition, they did not have to deal with the stress of having a mutilated body.

In this vein, the aim of the current study is to investigate the role of coping strategies in predicting emotional distress of breast cancer, over and above the illness severity - operationalized in terms of the type of surgery performed - traditionally associated with it. In order to achieve this goal, two groups of newly diagnosed breast cancer women were selected and compared on the basis of the type of surgical treatment received. Since the method of sentinel lymph-node biopsy (SLNB) has been introduced in the Breast Surgery Unit of “San Paolo” Hospital, partial removal of the breast and SLNB is the treatment of election, given a diagnosis of breast cancer at a very early stage and negative SLNB results. Prior to the intervention, in fact, women have a consultation with the surgeon and the breast specialist in order to decide the most suitable type of surgery according to the initial diagnosis. Sentinel lymph node biopsy should be the treatment of choice for patients who have early-stage breast cancer with clinically negative nodes. Compared with standard axillary treatment, sentinel lymph node biopsy was associated with reduced arm morbidity and better quality of life with no increase in anxiety (Mansel et al., 2006a, 2006b). In contrast to abstract information about tumor grade and other illness characteristics, the kind of surgery required has concrete meaning for the patients. In fact, they know that lymph node status is highly related to prognosis and to the likelihood of chemotherapy. For this reason, the necessity of mastectomy with axillary dissection was considered the most suitable indicator about disease severity from the patient’s perspective. It was assumed, therefore, that the women who had mastectomy and axillary dissection (MAD) have a perception of a higher severity compared to the patients with quadrantectomy and SLNB.

According to Lazarus (1993), coping refers to the intentional efforts in managing the sources of psychological stress. Investigating the role of responses to stress, following a breast cancer diagnosis, Glinder et al. (2007) and Yang, Brothers, and Andersen (2008) distinguish between ‘engagement’ versus ‘disengagement’ coping responses, being the former characterized by a psychological attitude of moving towards the stressor, in order to exert control through various cognitive and emotional strategies. Disengagement, in contrast, is characterized by “orienting away from the source of stress and one’s emotional responses. Examples of disengagement coping include cognitive and behavioral avoidance, suppression of unwanted thoughts and emotions, and denial” (p. 338). Other studies as well confirm that problem-focused strategies (engagement coping) are associated with decreased anxiety, decreased depression and less psychological distress (Bussell & Naus, 2010; Stanton, Danoff-Burg, & Huggins, 2002). Belief that breast cancer would have a chronic timeline is strongly related to the use of cognitive avoidance and behavioral avoidance coping strategies, with less use of problem-solving strategies (Hagger & Orbell, 2003; Rozema, Völlnick, & Lechner, 2009). We presume, therefore, that women who have a coping style more oriented towards avoidance will manifest higher distress.

## Aims and Hypotheses

The present study adopted a descriptive correlational design in order to examine the role of coping strategies in predicting psychological distress (anxiety and depression), over and above the illness severity. On the basis of the aforementioned literature, we operationalized the illness severity construct in terms of type of surgery (Mansel et al., 2006a, 2006b). We compared patients who had mastectomy and axillary dissection (MAD) vs. patients with quadrantectomy and SLNB. Based on findings of the above quoted literature, we expected that:

H1. Patients with MAD will exhibit higher levels of anxiety and depression than patients with SLNB.

H2. Avoidance coping strategies will be positively associated with higher levels of anxiety and depression.

H3. Engagement coping strategies will be negatively associated with higher levels of anxiety and depression.

H4. Coping strategies will explain statistically significant increment of variance in psychological distress (anxiety and depression), over and above type of surgery (SLNB vs. MAD).

## Method

### Participants

Women diagnosed with their first occurrence of breast cancer were eligible to selection for this study. Exclusion criteria were recurrence of breast cancer, previous or current cancer diagnosis, different from breast cancer, severe mental or neurological impairment. Patients were consecutive cases at the Breast Surgery Unit of “San Paolo” Hospital of Bari, during the year 2013.

Among all patients evaluated, only those who had fully completed both questionnaires were eligible. Of these, 30 women were treated with quadrantectomy and SLNB, and 31 patients were treated with MAD. All women were Southern Italian and resident in Puglia, primarily Bari and its Province (75%), middle aged (*M* = 54, *SD* = 11.90 years), married (75%).

### Measures

#### Coping

Coping strategies were assessed using the Italian version of Brief COPE - dispositional version, (Carver, 1997; Conti, 1999). It is a 28 item inventory that is a shorter version of the COPE Inventory (Carver et al., 1989). It includes 14 scales that correspond to 14 different coping strategy: a) Self-distraction (Cronbach’s alpha = .37), b) Active coping (Cronbach’s alpha = .55), c) Denial (Cronbach’s alpha = .59), d) Substance use (Cronbach’s alpha = .24), e) Use of emotional support (Cronbach’s alpha = .67), f) Use of instrumental support (Cronbach’s alpha = .73), g) Behavioral disengagement (Cronbach’s alpha = .76), h) Venting (Cronbach’s alpha = .23), i) Positive reframing (Cronbach’s alpha = .63), l) Planning (Cronbach’s alpha = .48), m) Humor (Cronbach’s alpha = .69), n) Acceptance (Cronbach’s alpha = .57), o) Religion (Cronbach’s alpha = .86), and p) Self-blame (Cronbach’s alpha = .19). Each scale has 2 items, each assessed on a four point Likert scale ranging from 1 = *‘I haven’t been doing this at all’* to 4 = *‘I’ve been doing this a lot’*. The scale scores define a “profile” for the subject’s coping style, which is how a subject usually responds to a stressor.

#### Psychological distress

The Italian version of the Hospital Anxiety and Depression Scale (HADS) (Costantini et al., 1999; Zigmond & Snaith, 1983) was used to measure distress. The HADS has been validated with cancer patients and it is very frequently used as screening tool for psychological distress in cancer care (Moorey et al., 1991; Smith et al., 2002). It consists of 14 items, rated on a 4-point scale, ranging from 0-3. Items were averaged into two dimensions: a) HADS-anxiety (Cronbach’s alpha = .81), b) HADS-depression (Cronbach’s alpha = .65).

### Procedure

The initial psychological assessment, performed during hospitalization, two days after surgery, consisted of a semi-structured interview followed by two self-report questionnaires: the HADS and the Brief COPE. All questionnaires were completed on the ward. All participants provided written, informed consent prior to taking part in the consultation. The first author, expert clinical psychologist, conducted all individual interviews that lasted an average of 45 minutes. A health status assessment was derived from both medical chart inspection and physician consultation.

Demographic data and general health status were recorded on a socio-demographic form. Ethical approval from local research ethics committee was obtained.

### Data Analyses

The independent-samples t-test and chi-square test of independence were run to assess that the two sub-samples were homogenous as regards demographic and medical characteristics. Descriptive and reliability analyses were run on HADS and Brief Cope indices. Cronbach’s alpha values obtained in our study for each dimension are reported above in the Measures section. The independent-samples t-test analysis was run to examine the surgery differences in terms of coping strategies and psychological distress. Zero-order Pearson’s correlation analyses assessed the associations between coping strategies and anxiety, depression, and separately for SLNB and MAD sub-samples. Hierarchical multiple regression analyses tested a) the extent to which the type of surgery and patients’ coping style predicted psychological distress, and b) the role of coping strategies in explaining an incremental variance in psychological distress, over and above the type of surgery.

## Results

### Sample Characteristics

The two groups (SLNB vs. MAD) were homogenous as regards age, *t*(59) = .72, *p* = .48, education distributions, χ2(3, *N* = 61) = 1.69, *p* = .64, marital status, χ2(3, *N* = 61) = 4.43, *p* = .22, employment status, χ2(6, *N* = 61) = 9.37, *p* = .15, general health (i.e., having chronic disease, such as hypertension, thyroid dysfunction, diabetes, etc.; χ2(3, *N* = 61) = 2.33, *p* = .51, and previous experiences of cancer in a close relative, χ2(4, *N* = 59) = 2.71, *p* = .61). As Table 1 shows, the major difference is in terms of occupation, which is also reflected in the total number of the cases examined in 2013 (*N* = 220), where housewives have higher percentage of mastectomy (50%) compared to working women (42%). It is possible that higher education and work play a role in preventive practices and early detection of breast cancer in our sample as well, as it is reported in other studies (Montella et al., 1995; Wang, Luo, & McLafferty, 2010). The demographic characteristics for the two subsamples are shown in Table 1.

**Table 1:** Demographics and Medical Characteristics of Participants

Characteristic	SLNB sample^a^		MAD sample^b^	
	*n*	%	*n*	%
Higher level of education >12 years	18	60	14	45
Marital status		
Single	4	13	4	13
Married	20	67	26	84
Separated/Divorced	1	3	0	0
Widowed	5	17	1	3
Employment status		
Housewife	9	30	17	55
Unemployed	0	0	1	3
Employed	17	57	10	32
Retired	4	13	3	10
Other chronic illnesses	21	70	18	58
Cancer in close relative		
Family of origin	13	43	15	48
Nuclear family (spouse or child)	2	7	0	0

^a^*n* = 30. Age: range = 40-78 years, *M* = 55.13 years, *SD* = 11.10 years.

^b^*n* = 31. Age: range = 26-80 years, *M* = 52.94 years, *SD* = 12.72 years.

### Descriptive Analyses

Table 2 displays the descriptive analyses outcomes of Brief Cope and HADS measures. Mean ratings showed that our sample used prevalently engagement coping strategies (especially acceptance, planning, religious support) than avoidance ones (especially substance use or behavioral disengagement; *F*(13,60) = 69.00, *p* = .001). Furthermore, in regard to HADS indices, mean ratings showed that patients reported medium level of anxiety and low level of depression. Generally, they showed higher anxiety than depression (dependent-samples *t*(59) = 10.88, *p* = .001). Additionally, the independent-samples t-test showed no difference between the two groups of patients in the coping strategies; instead women who had mastectomy exhibited higher levels of anxiety and depression than women who undertook SLNB (see Table 2). According to Cohen (1977), the effect sizes can be considered moderate or high.

**Table 2:** Descriptive Statistics and t-Test for Brief Cope and HADS

Measures	Total sample (*N =* 61)		SLNB sample (*n* = 30)		MAD sample (*n* = 31)		*t*(59)	*Cohen’s d*
	*M*	*SD*	*M*	*SD*	*M*	*SD*		
BC-Self-distraction	2.98	.64	3.03	.66	2.93	.63	.63	.16
BC-Active coping	2.98	.72	2.93	.78	3.03	.67	-.52	-.14
BC-Denial	1.68	.72	1.53	.64	1.83	.77	-1.66	-.43
BC-Substance use	1.06	.24	1.02	.09	1.10	.33	-1.29	-.12
BC-Emotional support	2.41	.74	2.33	.79	2.49	.69	-.81	-.22
BC-Instrumental support	2.49	.70	2.32	.72	2.66	.63	-1.96	-.51
BC-Behavioral disengagement	1.45	.61	1.35	.40	1.55	.80	-1.30	-.32
BC-Venting	2.34	.53	2.22	.50	2.46	.54	-1.81	-.47
BC-Positive reframing	2.74	.72	2.78	.80	2.70	.64	.43	.11
BC-Planning	3.15	.61	3.01	.71	3.29	.47	-1.82	-.47
BC-Humor	2.01	.77	1.85	.74	2.17	.78	-1.62	-.43
BC-Acceptance	3.24	.60	3.26	.65	3.22	.56	.24	.07
BC-Religion	3.10	.93	3.08	1.05	3.11	.80	-.12	-.03
BC-Self-blame	2.21	.69	2.21	.69	2.22	.70	-.05	-.01
HADS-Anxiety	1.41	.53	1.28	.57	1.54	.47	-1.98*	-.51
HADS-Depression	.83	.44	.67	.45	.98	.38	-2.97**	-.76

*p < .05. **p < .01.

### Correlations Between Coping Strategies and Psychological Distress

We conducted zero-order Pearson’s correlations to examine the associations between coping strategies, anxiety, and depression for total sample and separately for SLNB and MAD sub-samples (Table 3). The Bonferroni correction was applied to our multiple correlations (84 correlations; 14 Brief Cope measures with 2 HADS measures for three samples; Bonferroni corrected α = .05/84 = .001) to control the experiment-wise error rate. For the total sample, HADS-anxiety was positively associated with coping strategies of denial and venting, while depression appeared to be positively associated with behavioral disengagement and venting strategy. For the SLNB sub-sample, HADS-depression was positively associated with venting strategy, and negatively with acceptance coping strategy. For the MAD sub-sample, HADS-depression index was positively associated with Brief Cope-venting.

**Table 3:** Zero-Order Correlations Between Brief Cope and HADS Measures

Measures	Total sample (*N* = 61)		SLNB sample (*n* = 30)		MAD sample (*n* = 31)	
	HADS-A	HADS-D	HADS-A	HADS-D	HADS-A	HADS-D
BC-Self-distraction	-.13	-.16	-.11	-.08	-.12	-.21
BC-Active coping	-.10	-.07	-.15	-.16	-.09	-.03
BC-Denial	.37*	.27	.31	.11	.38	.32
BC-Substance use	.05	.23	.24	.38	-.06	.17
BC-Emotional support	.29	.16	.23	.10	.33	.18
BC-Instrumental support	.22	.20	-.04	-.11	.44	.42
BC-Behavioral disengagement	.05	.35*	.11	.39	-.05	.31
BC-Venting	.34*	.53*	.35	.50*	.25	.49*
BC-Positive reframing	-.22	-.21	-.26	-.38	-.15	-.16
BC-Planning	-.03	-.14	-.10	-.35	-.09	-.09
BC-Humor	-.27	-.03	-.24	-.16	-.47	-.06
BC-Acceptance	-.32	-.31	-.34	-.53*	-.31	-.06
BC-Religion	.04	.09	-.17	-.04	.35	.27
BC-Self-blame	.28	.23	.40	.36	.17	.11

*Note.* HADS-A = Hospital Anxiety and Depression Scale – Anxiety; HADS-D = Hospital Anxiety and Depression Scale – Depression.

*p < .01.

### The Prediction of Psychological Distress

To test a) the extent to which the type of surgery and patients’ coping style predicted psychological distress, and b) the role of coping strategies in explaining an incremental variance in psychological distress, over and above the type of surgery, we ran two hierarchical multiple regression model (HMR) analyses. For each HMR, the HADS index was the dependent variable (HADS-anxiety and HADS-depression), with age at Step 1, type of surgery at Step 2, and the fourteen Brief Cope strategies at Step 3.

For the HADS-anxiety index, as shown in Table 4, at both Step 1 and 2 the models and the incremental change are not significant (respectively, *F* = .65, *p* = .42; *F* = 2.15, *p* = .13, R^2^ change = .06, *p* = .06). At Step 3, when the fourteen Brief Cope strategies are introduced, the model and the incremental change are significant (*F* = 2.58, *p* = .007; R^2^ change = .42, *p* = .01), with emotional support, venting, and humor as the significant predictors.

**Table 4:** Hierarchical Multiple Regression of HADS-Anxiety

Measures	Step 1		Step 2		Step 3	
	β	*p*	β	*p*	β	*p*
Step 1						
Age	-.10	.43	-.08	.52	.00	.98
Step 2						
Surgery			.24	.06	.23	.09
Step 3						
BC-SE					-.09	.50
BC-AC					-.00	1.00
BC-DE					.19	.23
BC-SU					-.21	.16
BC-ES					.38	.04
BC-IS					-.13	.50
BC-BD					-.09	.53
BC-VE					.33	.05
BC-PR					.02	.91
BC-PL					.05	.75
BC-HU					-.36	.02
BC-ACC					-.14	.31
BC-RE					-.17	.23
BC-SB					.13	.37
*R^2^*	.01	.42	.07	.13	.48	.01
Δ*R^2^*			.06	.06	.42	.01

*Note:* BC-SE = Brief Cope – Self-distraction; BC-AC = Brief Cope – Active coping; BC-DE = Brief Cope – Denial; BC-SU = Brief Cope – Substance use; BC-ES = Brief Cope – Emotional support; BC-IS = Brief Cope – Instrumental support; BC-BD = Brief Cope – Behavioral disengagement; BC-VE = Brief Cope – Venting; BC-PR = Brief Cope – Positive reframing; BC-PL = Brief Cope – Planning; BC-HU = Brief Cope – Humor; BC-ACC = Brief Cope – Acceptance; BC-RE = Brief Cope – Religion; BC-SB = Brief Cope – Self-blame.

For the HADS-depression index, as shown in Table 5, at Step 1 the model is not significant (*F* = .58, *p* = .45). At Step 2, when the surgery is introduced, the model and the incremental change are significant (*F* = 5.02, *p* = .01; *R*^2^ change = .14, *p* = .003), with surgery as the significant predictor. At Step 3, when the fourteen Brief Cope strategies are introduced, the model and the incremental change are significant (*F* = 2.48, *p* = .009; *R*^2^ change = .33, *p* = .046), with surgery and venting as the significant predictors.

**Table 5:** Hierarchical Multiple Regression of HADS-Depression

Measures	Step 1		Step 2		Step 3	
	β	*p*	β	*p*	β	*p*
Step 1						
Age	.10	.45	.13	.28	.07	.62
Step 2						
Surgery			.37	.00	.29	.03
Step 3						
BC-SE					-.09	.53
BC-AC					.10	.54
BC-DE					-.06	.71
BC-SU					-.11	.44
BC-ES					.12	.50
BC-IS					-.01	.98
BC-BD					.23	.11
BC-VE					.38	.03
BC-PR					.00	.98
BC-PL					-.12	.48
BC-HU					-.11	.47
BC-ACC					-.16	.25
BC-RE					.01	.94
BC-SB					.13	.36
R^2^	.01	.45	.15	.01	.47	.00
ΔR^2^			.14	.00	.33	.05

*Note.* BC-SE = Brief Cope – Self-distraction; BC-AC = Brief Cope – Active coping; BC-DE = Brief Cope – Denial; BC-SU = Brief Cope – Substance use; BC-ES = Brief Cope – Emotional support; BC-IS = Brief Cope – Instrumental support; BC-BD = Brief Cope – Behavioral disengagement; BC-VE = Brief Cope – Venting; BC-PR = Brief Cope – Positive reframing; BC-PL = Brief Cope – Planning; BC-HU = Brief Cope – Humor; BC-ACC = Brief Cope – Acceptance; BC-RE = Brief Cope – Religion; BC-SB = Brief Cope – Self-blame.

Jointly considered, confirming our Hypothesis 4, coping strategies (especially emotional support, venting, and humor) explained a statistically significant increment of variance in psychological distress indices.

## Discussion

The present study was aimed at examining the role of coping strategies in predicting psychological/emotional distress of newly diagnosed breast cancer women, over and above the illness severity. Confirming Hypothesis 1, patients with MAD exhibited higher levels of anxiety and depression than patients with SLNB. Previous studies tended to find a relationship between the type of surgery (mastectomy vs. lumpectomy) and emotional distress, but were not able to demonstrate any significant difference, except for body image, in terms of the psychological effect of the type of mutilation (Bartelink, van Dam, & van Dongen, 1985; Cohen, Hack, de Moor, Katz, & Goss, 2000; Engel, Kerr, Schlesinger-Raab, Sauer, & Hölzel, 2004; Fallowfield, Baum, & Maguire, 1986; Hack, Cohen, Katz, Robson, & Goss, 1999).

Partially confirming Hypotheses 2 and 3, for the total sample, HADS-anxiety was positively associated with coping strategies of denial, venting, and behavioral disengagement, while depression appeared to be positively associated with venting strategy. For the SLNB sub-sample, HADS-depression was positively associated with venting strategy and negatively with acceptance coping strategy. For the MAD sub-sample, HADS-depression index was positively associated with Brief Cope-venting.

Finally, confirming Hypothesis 4, coping strategies will explain statistically significant increment of variance in psychological distress (anxiety and depression), over and above type of surgery (SLNB vs. MAD). More specifically, jointly considered, coping strategies (especially emotional support, venting, and humor) explained a statistically significant increment of variance in psychological distress indices. Most studies are based on the assumption that cognitive understanding of the illness has an influence on coping strategies and, as a consequence, on psychological and physical health (Keeling, Bambrough, & Simpson, 2013; McCorry et al., 2013). On the other hand, as it is reported in several meta-analysis, results from various studies about coping with breast cancer and illness severity are difficult to be compared due to the different assessment procedures and instruments utilized.

Taken together, the findings of the current study have important clinical practical implications for women with breast cancer: the dispositional coping strategies, that each individual has, appeared to be crucial to reduce distress and increase quality of life. Not only is the illness severity to account for the emotional distress, but, above all, the coping strategies play a significant role. Therefore, they should be considered as one of the most important factors when implementing effective interventions with breast cancer women. Encouraging engagement coping could contribute to improve patients’ emotional well-being.

However, our study is not without limitations. First, the reported findings are based on a mostly correlational study, so caution should be taken when inferring conclusions. Second, some Brief Cope indices showed low reliability values for the sample in our study (especially self-blame). Third, the small sample size does not allow for generalization. Fourth, neither patient’s level of control nor individual differences or personality traits (i.e. extroversion and/or neuroticism trait) were considered, beyond the coping styles.

Far from being exhaustive and despite its limitations, the current study adds to our knowledge of salient variables and processes that underlie health and psychological functioning in breast cancer settings, yielding some immediate consequences for clinical practice and future research.

Whilst the clinical setting is an asset of this study – it allowed, in fact, for control of the variables intrinsic to the specific situation in which the examination occurred, that was the same for both subgroups – it is, at the same time, a limit, in that the lack of a standardized coding for the clinical interview did not allow to use this information for comparison with self-report results.

On the research level, therefore, the integration of qualitative-observational and self-report methods needs to be thoroughly structured in order to obtain more reliable and objective results, arising from combined perspectives: a subjective evaluation form the patient’s side and a clinician’s evaluation. A more exhaustive assessment would imply to use the Brief Cope situational-actual version, in order to consider the coping strategies activated in response to the actual stressor (e.g., breast cancer diagnosis) and the evaluation of the emotional reactions and psychological distress through additional measures, besides HADS, such as BSI (Brief Symptom Inventory-18; Derogatis, 2001), IPQ-R (Illness Perception Questionnaire-Revised; Moss-Morris et al., 2002), and IES-R (Impact of Event Scale-Revised, Weiss & Marmar, 1997). Additionally, further studies may test an alternative modelling approach that would look at potential interaction terms of coping strategy by type of surgery on degree of emotional distress.

As the literature highlights, in fact, the main factor that predicts psychological morbidity across the 1-year follow up of women diagnosed with primary breast cancer is their immediate post-operative state of distress (Hack & Degner, 2004; Millar, Purushotham, McLatchie, George, & Murray, 2005; Vos, Garssen, Visser, Duivenvoorden, & de Haes, 2004). Therefore, on the clinical level, our study contributes to fostering the evaluation of psychological distress and of psychological resources (coping strategies) as crucial for implementing the development of tailored procedures, according to the woman’s specific needs and coping strategies, within the hospital program of psychological support for breast cancer patients.
